# Perforated carcinoma of the caecum presenting as necrotising fasciitis of the abdominal wall, the key to early diagnosis and management

**DOI:** 10.1186/1471-2482-6-11

**Published:** 2006-09-29

**Authors:** Conor D Marron, Gerarde T McArdle, Milind Rao, Stephen Sinclair, John Moorehead

**Affiliations:** 1 Department of Surgery, Ulster Hospital Dundonald, Upper Newtownards Road, Belfast, BT16 1RH, Northern Ireland, UK; 2 Department of Plastic Surgery, Ulster Hospital Dundonald, Upper Newtownards Road, Belfast, BT16 1RH, Northern Ireland, UK; 3 Department of Surgery, ICS, Royal Victoria Hospital, Grosvenor Road, Belfast, BT12 6BA, Northern Ireland, UK

## Abstract

**Background:**

Necrotising Fasciitis is a life threatening soft tissue infection which requires aggressive, early surgical management.

**Case presentation:**

We present a rare case of a retroperitoneal perforation of a carcinoma of the caecum presenting as a necrotising fasciitis of the anterior abdominal wall.

**Conclusion:**

This case highlights the importance of early aggressive debridement to healthy tissue limits, the consideration of a rare underlying cause, and the scope for plastic surgical reconstruction in order that aggressive initial surgery can be adequately performed.

## Background

Necrotising Fasciitis is a life threatening soft tissue infection which requires aggressive, early surgical management. The precise aetiology of necrotising fasciitis is unclear in many cases. We present a rare case of a retroperitoneal perforation of a carcinoma of the caecum presenting as a necrotising fasciitis of the anterior abdominal wall.

## Case presentation

A 52 year old male presented with a one week history of discomfort and swelling in the right iliac fossa, and 12 hours of skin erythema which was rapidly progressing. Within 2 hours of assessment, the cellulitis was noted to have spread outside the boundaries marked on admission. He was hypotensive, tachycardic, febrile and noted to have an elevated white cell count.

**Figure 1:**
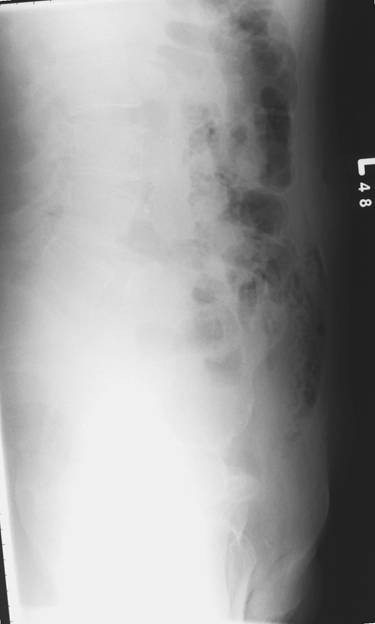
Lateral Abdominal X-Ray demonstrating subcutaneous gas within the abdominal wall.

On examination of the area there was diffuse oedema of the skin with marked erythema and heat in the area of skin from the right loin to groin across to midline. Subcutaneous crepitus was present. A lateral abdominal X-Ray revealed free gas within the subcutaneous tissues (Fig. 1).

A presumed diagnosis of necrotising fasciitis was made and the patient commenced on intravenous antibiotics, resuscitated with intravenous fluids, and taken immediately to the operating theatre.

**Figure 2:**
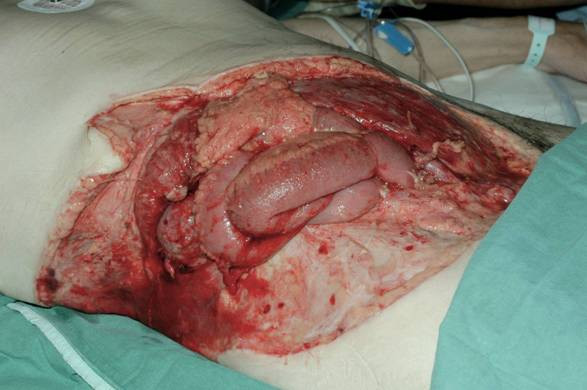
Extensive debridement of abdominal wall leaving small bowel and kidney exposed.

Extensive debridement of the affected cutaneous, subcutaneous tissue, and muscle was performed and pus noted to extend to the right loin area. Histology and bacteriology confirmed a diagnosis of necrotising fasciitis, with a β-haemolytic group streptococcus cultured. Exploration revealed a retroperitoneal abscess arising as a result of a perforation of a caecal carcinoma. The caecum was resected and a right hemicolectomy performed, with a large defect resulting in the anterior abdominal wall from the extensive debridement (Fig. 2).

**Figure 3:**
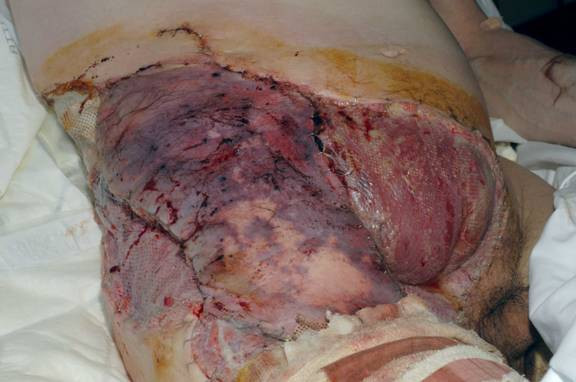
Defect in abdominal wall closed using rectus femoris myocutaneous flap.

The patient was admitted to the intensive care unit for inotropic support and management of sepsis, and 2 days later proceeded to have a rectus femoris myocutaneous flap fashioned to close the defect in the abdominal wall. He made a good post-operative recovery, with adequate skin closure on the abdominal wall (Fig. 3), and was discharged from hospital 8 weeks post-operatively. The patient subsequently died at home, from a myocardial infarction, five months after surgery. Informed consent for publication and use of images had been received from the patient and his family.

## Discussion

Retroperitoneal, or psoas, abscess is an unusual problem, which has insidious and occult characteristic[1]. There are often delays in the diagnosis of retroperitoneal abscess due to its insidious nature, and it frequently extends beyond the peritoneum before being identified. Even in those cases with contamination from the gastro-intestinal tract, the course appears relatively benign. Retroperitoneal abscess rarely results from perforation of the colon as most perforations occur into the peritoneal cavity[1]. Those that perforate into the retroperitoneum rarely result in formation of a psoas abscess[1].

Conversely, necrotising fasciitis is a soft tissue infection associated with high morbidity and mortality[2]. Necrotising soft tissue infections have been recognised and reported for centuries the earliest dating back to Hippocrates in the 5^th ^century BC[3]. A rapidly progressive soft tissue infection (synergistic undermining gangrene) caused by the synergistic action of a β-haemolytic group streptococcus +/- staphylococcus, was described in 1924 by Meleney[4]. Wilson subsequently re-termed this observed phenomenon as necrotising fasciitis, in 1952[5].

Necrotising fasciitis is rare within the UK with an estimated at 500 new cases each year, however this is difficult to confirm, as different eponyms are given to describe the same condition, as outlined above[6]. The aetiology of necrotising fasciitis is not fully understood, with patients often having a history of trauma, including insect bites, scratches, or abrasions[7]. However in some cases no primary cause can be found. Patients that have pre existing conditions which increase susceptibility to infection seem to be at an increased risk of developing necrotising fasciitis these conditions include diabetes mellitus, peripheral vascular disease, chronic renal failure, drug misuse, and advanced age[8].

The cornerstone of management of necrotising fasciitis is recognised as being aggressive surgical debridement and intensive support, whilst other severe soft tissue infections do not necessarily require the same amount of aggressive debridement. Therefore it is important that clinicians are able to differentiate between the two.

Necrotising fasciitis should be considered in those patients who have rapidly progressing cellulitis, and palpable subcutaneous crepitus, in association with tachycardia, hypotension, neutropenia or neutrophilia, and hyponatraemia[6].

Adjuvant diagnostic tools for necrotising fasciitis include CT scanning and plain radiography[8]. In this case the clinical finding of crepitus, and soft tissue air on plain radiograph were both present, however these signs are only seen in 37% and 57% of patients respectively[6].

The finding of necrotising fasciitis of the anterior abdominal wall associated with a carcinomatous perforation of the colon is rare, having only previously been described with perforation of a sigmoid colon carcinoma directly adherent to the anterior abdominal wall[9]. In addition, this case is unusual in that retroperitoneal abscesses have been described to track along psoas, or through the obturator foramen to give swellings in the thigh or gluteal region, but not to give rise to infection of the anterior abdominal wall[1,9].

This case illustrates that the principles of management should be two-fold; 1) To rapidly and unequivocally control infection and sepsis through extensive radical debridement of necrotic tissue and; 2) Identify and treat any underlying condition giving rise to the infection, in this case removing the caecal tumour.

Intravenous antibiotics should be started promptly and modified when sensitivities return. However, it is essential to ensure that adequate necrotic material is removed at the first opportunity to reduce the risk of further progression, regardless of the defect that will remain. Defects may be closed using a variety of techniques, including split skin grafts, Vacuum Assisted Closure (VAC) therapy[6], and flap procedures, including myocutaneous flaps [10-12]. Hyperbaric oxygen therapy in the treatment of necrotising fasciitis remains controversial, conflicting studies have reported contradicting results as to potential benefits[13].

## Conclusion

Early recognition of important signs of necrotising fasciitis is essential to effective management of this condition, and clinicians assessing patients with rapidly progressive cellulitis should be aware of these vital features.

In cases presenting with necrotising fasciitis there should be consideration given to an underlying cause for the infection.

Aggressive surgical management is the cornerstone to management of this condition, and is the key to survival for these patients. This case demonstrates that extensive debridement can be performed with resulting large defects, which can be adequately closed at a later time, and therefore this should not be thought of as a barrier to adequate surgical debridement and definitive treatment.

## Competing interests

The author(s) declare that they have no competing interests.

## Authors' contributions

All authors have read and approved the final version of the manuscript.

CM was involved in the conception for the idea for the report, and was involved with the diagnosis, operative, and post-operative management of the patient. He is responsible for preparation of the manuscript for submission

GMcA was responsible for up to date literature review, re-writing of manuscript prior to submission, gaining of consent for use of images, and management of the patient.

MR was responsible for ward care of the patient, review, and re-writing of manuscript prior to submission.

SS was the senior consultant Plastic Surgeon responsible for the patients operative management and post-operative care and was involved in review and alteration of the manuscript prior to submission.

RM was the Senior Consultant General Surgeon involved with operative and post-operative management of the patient and was responsible for critical review of the manuscript prior to submission.

## Pre-publication history

The pre-publication history for this paper can be accessed here:
